# Supplementary material for: The influence of horizontal violence on intention to leave among Jordanian nurses: A cross-sectional study
Source: PLoS One. 2024 Nov 11;19(11):e0307799. doi: 10.1371/journal.pone.0307799 (PMC11554039; doi:10.1371/journal.pone.0307799)
Supplement: Attachment — Submitted filename: Response to Editor and Reviewers.docx [file pone.0307799.s002.docx]

Response to Editor and Reviewers

Dear Editor,

Thank you for your effort and time spent on our submission.

We revised the manuscript and considered all comments from the reviewers. Also, the comments were very minor revision we reread the manuscript again and enhanced the writing as much as possible. We hope this time the decision will be positive.

Kind regards

Dear Reviewers,

Thank you for your time and effort of reading and commenting on our manuscript. We would like also to thank you for the supportive message that you written in the comments about the topic and its importance to conduct research.

All your comments were carefully considered and amended on the track changes file and then accepted on the manuscript file too.

We hope this will be satisfactory and will take our submission to the next step.

Regards
