# Supplementary material for: The influence of horizontal violence on intention to leave among Jordanian nurses: A cross-sectional study
Source: PLoS One. 2024 Nov 11;19(11):e0307799. doi: 10.1371/journal.pone.0307799 (PMC11554039; doi:10.1371/journal.pone.0307799)
Supplement: Attachment — Submitted filename: Horizontal Violence and intention to leave.docx [file pone.0307799.s003.docx]

**Horizontal Violence and intention to leave**

**Abstract part**

**Please don’t describe the aim or objectives of the study in a separate heading, but it is better to incorporate them in the background section of the abstract. Usually, after describing the gaps to be studied,. Better to describe briefly the how the study was performed and samples are selected (brief methodological methods).**

The title of the study described the **influence of horizontal violence on intention to leave among Jordanian nurses, but the background section states that** no study in Jordan has examined the influence of horizontal violence on **intent to stay** among nurses. The intentions to **leave** and **stay** are two opposing ideas, so please make it clear what outcome variable you want to measure. The result section of an abstract state that the findings showed that, **horizontal violence** is **moderate** among nurses in Jordan. What is your evidence to say moderate, severe, and mild. I have not seen any classification mechanism in your study.

**Introduction part**

**Incorporating some key literature is good, but they are large, written separately, and only describe about one study. Please make it brief in one paragraph.**

**It is better to incorporate the purpose and significance of the study into a short statement.**

**Methods**

**Please add the word "materials." and rewrite it as methods and materials**

**Please briefly describe how each study participant was selected.**

**Briefly describe the study areas (including how many nurses are there in each hospital and how many of them participated in the study).**

**There is no study period in the method part.**

**Please list the inclusion and exclusion criteria under a separate subheading.**

**Briefly describe the source and study population.**

**Please operationalize statistically words like intent to leave, horizontal violence, etc.**

**Describe your dependent and independent variables separately.**

**On page 7, the heading stated that ‘procedure’—what procedure does it indicate?**

**Result**

**There is no need to describe the data collection period in the result section, but in the method part. And the same for the variables section.**

**Was an independent t-test appropriate for such large samples?**

**Briefly describe the statistical models that you used in the method part**

**Overall, the title is good, but the rest (the body) of the manuscript does seem to be research, but it seems to be a report.**
